# Artificial Intelligence in Cardiology: Why So Many Great Promises and Expectations, but Still a Limited Clinical Impact?

**DOI:** 10.3390/jcm12072734

**Published:** 2023-04-06

**Authors:** Gian Luigi Nicolosi

**Affiliations:** Division of Cardiology, Policlinico San Giorgio, 33170 Pordenone, Italy; gianluigi.nicolosi@gmail.com

## 1. Introduction

Looking at the extremely large amount of literature, as summarized in two recent reviews on applications of Artificial Intelligence in Cardiology, both in the adult and pediatric age groups, published in the *Journal of Clinical Medicine* [1,2], we can easily affirm that the future of AI in this field should be bright and extremely helpful and fruitful, both for cardiologists and patients. We can then expect that the implementation of AI to different aspects of cardiology discipline will induce great advantages, improvements, and important innovations, which could have a great impact on daily practice. All this would include multi-modality imaging improvements, better digital infrastructures, fundamental support to diagnostic, therapeutic, and prognostic techniques and clinical approaches, and to the clinical decision-making process, leading to optimized organizations and procedures [1,2]. Integrating AI into cardiology practice is a change that the profession will embrace. In fact, AI has the potential to support physicians’ knowledge and decisions, towards a precision cardiology and to a more efficient, and hopefully also more efficacious, health care [1,2]. Despite this apparent tremendous potential, the impact of AI in current clinical cardiology practice is still limited [1,2]. In recent years, the *Journal of Clinical Medicine* has published several contributions in this field, which are the subject of this Editorial and could help to better understand the increased interest of the scientific community in the application of AI in cardiology, but also the difficulties and obstacles to be overcome.

## 2. AI in Cardiovascular Imaging

Artificial intelligence has been widely applied in the field of cardiovascular imaging, including echocardiography, cardiac computed tomography, cardiac magnetic resonance, and nuclear imaging [1,2].

In the echocardiographic field, AI may improve imaging quality, guiding scanning, and assisting in segmentation, processing, and analysis [1,2,3,4,5]. AI can help in view interpretation and classification, in the quantification of both cardiovascular structure and function, and in detecting wall motion abnormalities [1,2,3,4,5]. AI can also help differentiating physiological hypertrophy in athletes from hypertrophic cardiomyopathy, and in the identification and assessment of amyloidosis, pulmonary artery hypertension, and valvular heart disease, as mitral regurgitation and aortic stenosis [1,2,3,4,5]. 

Concerning stress echocardiography, a complex protocol has been reported where one project will be devoted to cardiac strain and artificial intelligence to establish the transition from the qualitative naked eye to the quantitative automated assessment of regional wall motion in order to solve the current limitation of strain inter-vendor variability and to consider segmental heterogeneity during stress. In particular, artificial intelligence potentially provides a solution for the automated and in-depth handling of imaging information, by making measurement objective [6,7].

In the field of cardiovascular magnetic resonance, AI has been shown to be helpful in reducing the time of image acquisition and analysis and in the automatic correction of artifacts [1,2,8]. The use of AI techniques in image segmentation has allowed also an automatic and accurate quantification of the volumes and masses of the left and right ventricles, with an occasional need for a manual correction. Furthermore, AI can be a useful tool for assessing myocardial tissue characterization and to directly help the clinician in the diagnosis and derivation of the prognostic information of cardiovascular diseases [1,2,8]. 

## 3. AI Support in Electrophysiology

An AI-based algorithm has been developed and validated, using a neural network, to identify the location of the accessory pathway (AP) in 357 consecutive Wolff–Parkinson–White (WPW) syndrome patients, based on the delta-wave polarity in the 12-lead ECG [9]. AI identified the correct AP location with an accuracy of 85.7% (95% CI 79.6–90.5, *p* < 0.0001) [9]. This was better than the predictive accuracy of the established ECG-based algorithms by Arruda, Milstein, and Fitzpatrick, which yielded a predictive accuracy of 53.2%, 65.6%, and 44.7%, respectively [9].

AI could also be useful for identifying atrial fibrillation mechanisms at genetic, cellular, organ, and patient levels, involving clinical, demographic, metabolic, and genomic aspects [10]. Future tailored approaches may integrate mechanistic markers at all these biological levels, using machine learning and the AI approach, to develop individualized models of AF onset, progression, and response to therapy [10]. This is in order to achieve a real precision and personalized medicine in atrial fibrillation patients [10]. 

## 4. AI Support in Clinical Care

A frequent monitoring approach, using a remote wearable wireless patient monitoring system and advanced bioinformatic tools, showed early cardiovascular changes among 492 hospitalized COVID-19 patients [11]. Big data analysis was conducted using advanced AI and bioinformatics tools [11]. This may serve to improve the early detection of the clinical deterioration of COVID-19 patients [11]. 

Artificial Intelligence can also be of great value in both cardio and cerebrovascular diseases in several important fields of interest, as in disease diagnosis and patient monitoring, in preventive care by scanning through images and reports, in risk stratification for primary or secondary prevention, and in resource and workflow optimization by leveraging administrative data [12]. 

## 5. AI Support in Disease Diagnosis and Risk Prediction

A Feasible Artificial Intelligence with Simple Trajectories for Predicting Adverse Catastrophic Events (FAST-PACE) solution for preparing immediate intervention in emergency situations was introduced [13]. FAST-PACE utilizes a concise set of collected features to construct an AI model that predicts the onset of cardiac arrest or acute respiratory failure from 1 h to 6 h prior to its occurrence. Data from the trajectory of 29,181 patients in intensive care units of two hospitals included periodic vital signs, a history of treatment, current health status, and recent surgery [13]. Only simple clinical traits obtained from 1 h to 6 h prior to adverse events were utilized to accurately predict acute cardiac arrest or respiratory failure. This suggests that a monitoring alert system and life-saving strategy can be implemented shortly before an adverse event [13]. 

Machine learning makes it possible to utilize basic laboratory parameters to generate a distinct cardiac-amyloidosis-related heart failure profile compared with cardiac-amyloidosis-unrelated heart failure patients [14]. This proof-of-concept study opens a potential new avenue in the diagnostic workup of cardiac amyloidosis and may assist physicians in clinical reasoning and decision making [14]. 

A study proved the feasibility of a deep-learning-based approach of a fully automated adipose tissue analysis in clinical cardiac CT and confirmed, in a large clinical cohort of 966 patients with intermediate Framingham risk scores for coronary artery disease, referred for coronary calcium scans, that the volume and attenuation of epicardial and paracardial adipose tissues are not correlated with coronary artery calcium score [15]. Broadly available deep-learning-based rapid and reliable tissue quantification should thus be discussed as a method to assess supplementary risk predictors in cardiac CT [15]. 

The clinical management of dilated cardiomyopathy patients is challenging given the large heterogeneity in disease phenotype, genetic background, and progression of disease. Interoperable big data infrastructures comprising electronic health records, registries, and other patient databases can now be used with new techniques, such as deep and machine learning, in order to identify phenotype clusters, assess new features that classify dilated cardiomyopathy phenotypes, and predict disease outcome and validate them across different international cohorts [16]. As technology advances, in this context, wearable devices provide exciting new opportunities to personalize care and move towards patient-tailored predictive and preventive medicine [16].

Using the database of the Korean National Health Insurance Service, 2,037,027 participants with hypertension were identified [17]. A deep learning model could accurately predict cardiovascular-disease-related hospitalization and death within a year in these patients [17]. The findings of this study could allow for prevention and monitoring by allocating resources to high-risk patients [17]. 

The performance of machine learning algorithms (MLA) and physicians in predicting left ventricular systolic dysfunction (LVSD) from a standard 12-lead ECG were compared by utilizing a dataset of 13,820 pairs of ECGs and echocardiography [18]. A deep residual convolutional neural network was trained for predicting LVSD (ejection fraction (EF) < 50%) from ECG. The ECGs of the test set (*n* = 850) were assessed for LVSD by the MLA and six physicians [18]. The inter-observer agreement between the physicians for the prediction of LVSD was moderate (κ = 0.50), with an average sensitivity and specificity of 70% [18]. The C-statistic of the MLA was 0.85. Repeating this analysis with LVSD defined as EF < 35% resulted in an improvement in the physicians’ average sensitivity to 84%, but their specificity decreased to 57% [18]. The MLA C-statistic was 0.88 with this threshold [18]. However, the performance of MLA does not seem too strong in this study and further research is needed to identify the unknown parameters used by MLA for the classification of ECGs due to the inherent “black box” feature of MLA.

A novel investigation of deep learning (DL) solutions for predicting cardiovascular disease (CVD) and stroke risk in diabetic foot infection (DFI) patients has been reported [19]. The Preferred Reporting Items for Systematic reviews and Meta-Analyses (PRISMA) search strategy was used for the selection of 207 studies [19]. This review focused on how a DFI may contribute to the already complex nature of CVD and stroke. An artificial-intelligence-based model for predicting the risk of CVD and stroke in DFI patients was described using the AI framework [19].

## 6. Why Still a Limited Clinical Impact of AI in Cardiology? Difficulties and Obstacles to Be Overcome

Some difficulties seem to derive from the apparent relative “isolation” of several AI experiences. In fact, some AI projects appear as mainly driven by relatively few dedicated specialists, both from the computer/engineer and the medical sides, who are deeply involved in their own scientific design. The many difficulties for the development and the implementation of AI projects are then easily inducing such specialists, to be successful, to concentrate their efforts to specifically identified limited and focused goals, to be developed inside the borders of their own or of few other selected institutions, or to a single department or a single hospital. This can carry the risk of producing published scientific literature, with a sometimes limited subsequent implementation in clinical practice and in different environments. 

On the other hand, generalizable results of any AI approach and the possibility of a subsequent more universal implementation and the acceptance of AI algorithms depend probably on a methodology of early involvement, possibly through a developed and organized dedicated digital infrastructure, of a network of several institutions and hospitals with a different complexity and type of referrals. Given the initial development of the AI project, this could allow a consideration of all the different concurrent scientific needs and different point of views of involved professionals, and even possibly of patients. The results could have then a higher probability to be accepted as useful, and then utilized, implemented and continuously updated by all the professional people involved and inside the entire network of involved institutions, and even peripheral hospitals. A large and close collaboration and training among computer scientists, clinical investigators, clinicians, other healthcare professionals, regulatory authorities, health providers, and possibly even patients and the general public, could also allow the identification more easily of the most relevant problems to be solved along each AI project [1,2]. 

Some of these problems are related to the certification of AI products as medical devices, to the privacy protection, to legislative and legal and responsibility issues in the case of derived wrong medical decisions, to the transparency and the physiological plausibility of AI results [1,2].

Some other risks and problems can derive also from the relatively small size or limited and too homogeneous training dataset (developing the model), which is not representative of the greater heterogeneity of the real world, running the risk of over-fitting. The training dataset should be different from the testing dataset, while the AI algorithm should hopefully continue to improve its behavior and performances during implementation, to be more universally trusted, accepted, and integrated as a routine helpful support in clinical practice [1,2]. 

AI algorithms should probably also be considering the potential heterogeneity of the real-world input structured and unstructured dataset of a different quality and different presentation and storage support, either analogical and/or digital, with sometimes variable and unexpected missing or incomplete data, deriving from professional and medically certified machines but also from wearable sensors and different devices. Natural language processing for the autonomous input of data, starting from free texts and unstructured reports, should also be considered. The important issues of open free access AI algorithms and cyber security should also be examined. Unbalanced involvement and responsibilities (intellectual properties, patented products, conflicts of interest) among engineers and physicians of different institutions should be avoided, or at least clarified. Adequate budget and investments should be planned to help to overcome the limited vision and limited goals in AI projects. 

A clear differential classification of the complexity of AI algorithms and products should help to recognize and overcome specific difficulties and problems. Implementation should be then easier for focused AI products, as for supporting image quality, scanning, recognition, classification, and assessment in multimodality imaging, while it could be more complex, requiring more steps, for algorithms supporting clinical care and decision, risk assessment, and diagnosis. 

The results of AI algorithms should be always compared with the more traditional statistical and usual approach and usual care, through a cost efficiency, but also a cost efficacy analysis, in order to avoid the overestimation of AI results. In effect, some AI results could appear, as only marginally superior and with marginal gain and not fully competitive, as compared with the results of other current usual approaches. On the other hand, these same results could be mostly helpful for the clinical and decision support of physicians and health personnel operating in small institutions, even those which are peripherally located, with a low complexity of referral and less easy access to more complex diagnostic facilities.

A final problem and difficulty could also derive from an underestimation or superficial and quick analysis of the limitations and possible drawbacks of some AI approaches. This should be solved by accepting to continuously analyze and discuss, widely and in deep detail, all the difficulties and obstacles which can be encountered in each AI project. On the other hand, it should be taken into account that the acceptance of this opportunity of a free and open thorough discussion and comparison with all the professionals interested, and also potentially involved in the project, could help to design an organized cooperative plan for the further continuous development of AI projects and to overcome inherent difficulties and obstacles. 
